# Dual Role of Fas/FasL-Mediated Signal in Peripheral Immune Tolerance

**DOI:** 10.3389/fimmu.2017.00403

**Published:** 2017-04-05

**Authors:** Akiko Yamada, Rieko Arakaki, Masako Saito, Yasusei Kudo, Naozumi Ishimaru

**Affiliations:** ^1^Department of Oral Molecular Pathology, Tokushima University Graduate School of Biomedical Sciences, Tokushima, Japan

**Keywords:** Fas, FasL, T cell, B cell, apoptosis, activation-induced cell death, autoimmunity, immune tolerance

## Abstract

Fas-mediated apoptosis contributes to physiological and pathological cellular processes, such as differentiation and survival. In particular, the roles of Fas in immune cells are complex and critical for the maintenance of immune tolerance. The precise pathways and unique functions associated with Fas/FasL-mediated signaling in the immune system are known. The dual character of Fas/FasL-mediated immune regulation that induces beneficial or harmful effects is associated with the onset or development of immune disorders. Studies on mutations in genes encoding Fas and FasL gene of humans and mice contributed to our understanding of the pathogenesis of autoimmune diseases. Here, we review the opposing functions of Fas/FasL-mediated signaling, bilateral effects of Fas/FasL on in immune cells, and complex pathogenesis of autoimmunity mediated by Fas/FasL.

## Introduction

Fas receptor (CD95, tumor necrosis factor receptor superfamily member 6) is a death receptor (DR) localized on the surface of various cells, which triggers a signal transduction pathway leading to apoptosis (1, 2). The interaction of Fas with its ligand FasL (FasL/CD95L) regulates numerous physiological and pathological processes that are mediated through programmed cell death (3). The beneficial and harmful effects of Fas-mediated apoptosis on the immune system were identified after the discovery (4–6). Moreover, signaling downstream of Fas is intricately regulated by numerous pathway components (7–9).

MRL-*lpr/lpr* mice bear mutations in the gene encoding Fas and serve as a widely used model for autoimmune diseases, such as systemic lupus erythematosus (SLE), rheumatoid arthritis (RA), Sjögren’s syndrome (SS), and autoimmune lymphoproliferation syndrome (ALPS) (10, 11). Fas-mediated apoptosis of peripheral T cells, which represents a key mechanism that maintains immunological tolerance, is impaired in MRL-*lpr/lpr* mice. This mechanism, known as activation-induced cell death (AICD), deletes overactivated or autoreactive T cells in the periphery (12). The deletion of peripheral T cells by AICD is impaired in MRL-*lpr/lpr* mice, leading to increased autoreactive T cells that trigger the induction of autoimmune lesions in multiple organs (10, 13). Moreover, mutations in the gene encoding Fas occur in patients with ALPS (14, 15).

By contrast, FasL expression on the cell surface is specific to the immune system. For example, FasL expression by T cells is associated with their activation (4). FasL is unstable because it is shed from the cell surface through the action of certain enzymes (16, 17). When soluble FasL (sFasL) binds to Fas, cell proliferation, but not apoptosis, is induced (18). Mice with the *gld/gld* genotype bear mutations in the gene encoding FasL, and they are widely used as a model of autoimmune disease (19, 20). Moreover, mutations of the gene encoding FasL occur in patients with ALPS (14, 15, 21), and FasL is expressed by thyroid, endothelial, and corneal cells (22–24). Expression of FasL by cells residing in “immunoprivileged site” protects them from attack by activated or autoreactive lymphocytes (5).

This review describes the multiple functions of Fas/FasL in the immune system with focus on duality of Fas/FasL signaling in immune regulation and autoimmunity.

## Fas-Mediated Apoptosis

Fas protein has 319 amino acids and the predicted molecular weight is 48 kDa. The mature protein is divided into three domains: an extracellular domain, a transmembrane domain, and a cytoplasmic domain. The extracellular domain consists of 157 amino acids with cysteine-rich domain. The transmembrane and cytoplasmic domains have 17 and 145 amino acids, respectively. Exons 1 through 5 encode the extracellular region. Exon 6 encodes the transmembrane region. Exons 7–9 encode the intracellular region (1, 2).

**Figure 1:**
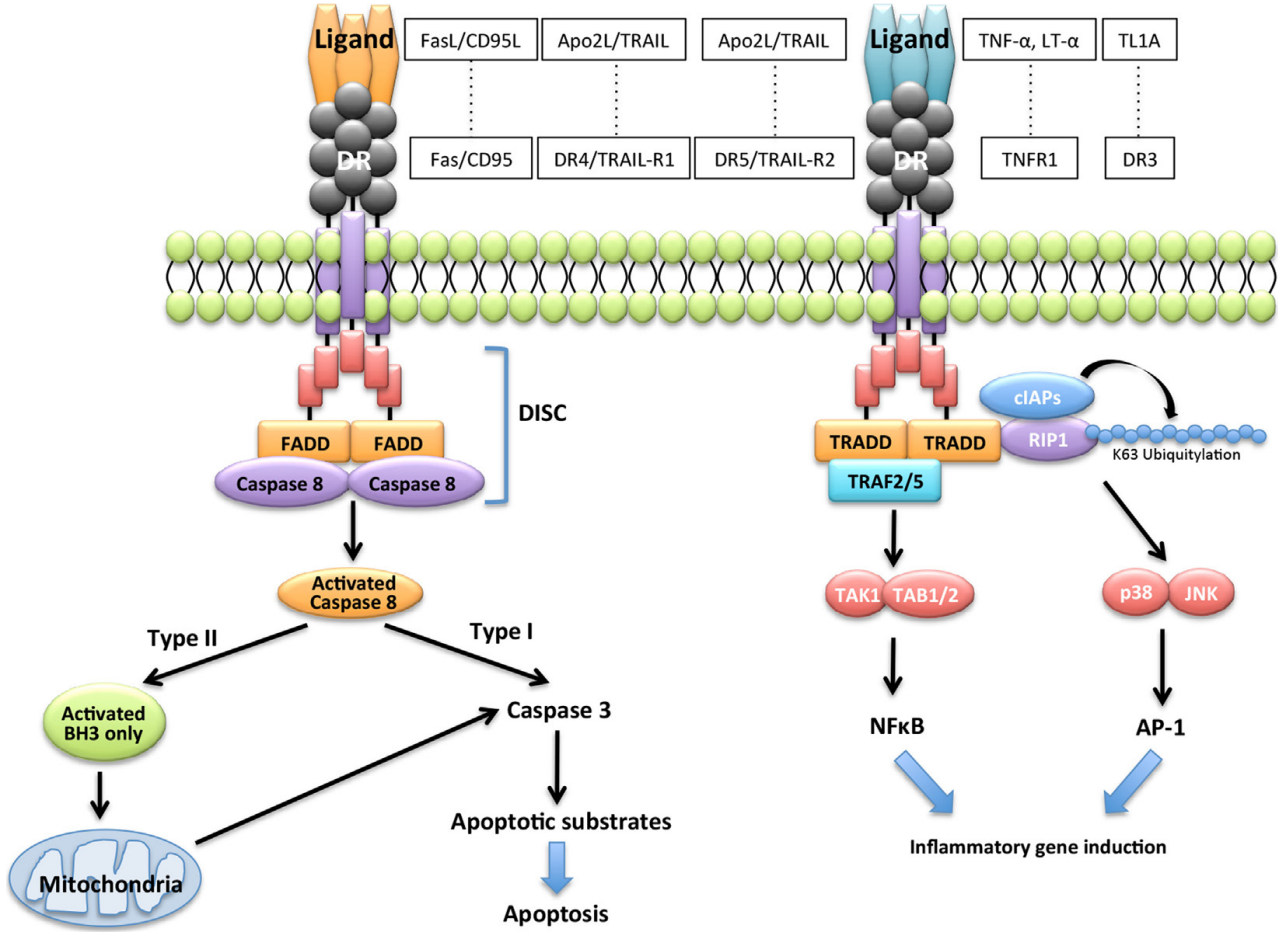
**Apoptotic signaling *via* Fas/FasL**. Engagement of the death receptors (DRs) with their cognate ligands, such as FasL/CD95L, tumor necrosis factor-alpha (TNF-α), lymphotoxin-alpha (LT-α), TNF-like protein-1A (TL1A), and Apo2L/TNF-related apoptosis-inducing ligand (TRAIL), and their receptors promotes recruitment and activation of the apoptosis-initiating proteases, caspase-8, and caspase-10 and induces transcriptional events leading to nuclear factor-kappa β (NF-κB)-dependent pro-inflammatory cytokine expression.

Extrinsic and intrinsic pathways are the major DR-mediated pathways of apoptosis. Engagement of the DRs with cognate ligands including FasL induces recruitment and activation of the apoptosis-initiating proteases, such as caspase-8 and caspase-10, and then induces apoptosis through various molecules. By contrast, the binding of some cognate ligands to DR triggers transcriptional events leading to nuclear factor-kappa β (NF-κB)- or activator protein-1 (AP-1)-dependent pro-inflammatory cytokine expression (Figure 1). DRs, which are members of a subset of the TNF receptor superfamily known as death receptors, possess a cytoplasmic death domain (DD). Fas-mediated apoptosis proceeds through the extrinsic pathway *via* the binding to their respective receptors of ligands, such as FasL, tumor necrosis factor-alpha (TNF-α), lymphotoxin-alpha (LT-α), TNF-like protein-1A (TL1A), and Apo2L/TNF-related apoptosis-inducing ligand (TRAIL) (Figure 1). FasL is the ligand for the Fas receptor. TNF-α and LT-α are ligands for the TNF superfamily member 1A (TNFR1), TL1A is a ligand for TNF receptor superfamily member 25 (DR3), and TRAIL is a ligand for the TNF receptor superfamily member 10a (DR4/TRAIL-R1) or tumor necrosis factor receptor superfamily member 10b (DR5/TRAIL-R2). These receptors are members of a subset of the TNF receptor super family known as DRs. Engagement of DRs of their cognate ligands promotes recruitment and activation of the apoptosis-initiating proteases caspase-8 and caspase-10 within membrane receptor complexes (25). Apoptosis induced by the engagement of the DRs by their cognate ligands is well understood (26–29) (Figure 1).

Binding of DRs to their cognate ligands recruits one of two pivotal DD-containing adaptor proteins: Fas-associated protein with DD (FADD) or TNF receptor-associated DD (TRADD) (Figure 1). FADD controls cell death by recruiting caspase-8 and caspase-10, TRADD controls non-apoptotic functions by recruiting the DD-containing kinase receptor-interacting protein-1 (30), and the E3 ubiquitin ligases TNF receptor-associating factor 2 (TRAF2) and cellular inhibitor of apoptosis proteins (31). TRADD signaling activates phosphorylation cascades comprising the IKK kinase complex, which phosphorylates the inhibitor of NF-κB and the mitogen-activated protein kinases, c-jun N-terminal kinase (JNK), and p38 (Figure 1). The recruiting events initiate a transcriptional program to express genes that induce the synthesis of mediators of inflammation (7–9). DRs can be divided into two categories based on the primary adaptor protein they bind to Fas/CD95/APO-1, DR4/TRAIL-R1 and DR5/TRAIL-R2 bind FADD and mediate mainly proapoptotic functions (Figure 1). By contrast, TNFR1 and DR3 bind TRADD and mediate mainly pro-inflammatory and immune-stimulatory activities (7–9) (Figure 1).

In the Fas-mediated apoptotic pathway, binding of FasL drives Fas clustering and binding of Fas to FADD. FADD recruits caspase-8 and caspase-10 to form the death-inducing signaling complex (DISC) (25). DISC is activated by specific post-translational modifications of the DR, such as palmitoylation and O-linked glycosylation (32, 33). The DISC mediates autocatalytic processing and activation of caspase-8 and caspase-10, which propagate the death signal either through proteolysis of effector caspases such as caspases-3, caspase-6, and caspase-7. In type I cells such as thymocytes, processing by effector caspases is sufficient to induce apoptosis (Figure 1). By contrast, apoptosis requires caspase-8-mediated cleavage of BH3-interacting domain death agonist (Bid), which is a BH3-only protein that can promote the permeabilization of mitochondrial outer membranes and release of cytochrome *c* in type II cells such as B cells (Figure 1). Upon release from mitochondria, cytochrome *c* acts as a cofactor for the assembly of a cytosolic caspase-activating complex called the apoptosome, which propagates the caspase activation cascade (33). Thus, Fas signal has dual pathways of apoptosis and non-apoptosis in various cells.

## Fas-Mediated T Cell Immune Regulation

The negative selection of autoreactive T cells in thymus is regulated by strong T cell signals that induce apoptosis through Bcl-2-interacting mediator of cell death (Bim), but not Fas (34). By contrast, apoptosis of activated peripheral T cells is controlled by the intrinsic and extrinsic pathways (34). Antigen stimulation *via* the T-cell receptor (TCR) contributes to antigen-specific T cell responses related to cell survival. TCR and Fas signaling are linked, and the AICD of peripheral T cells is controlled by interaction between TCR and Fas signaling (34).

**Figure 2:**
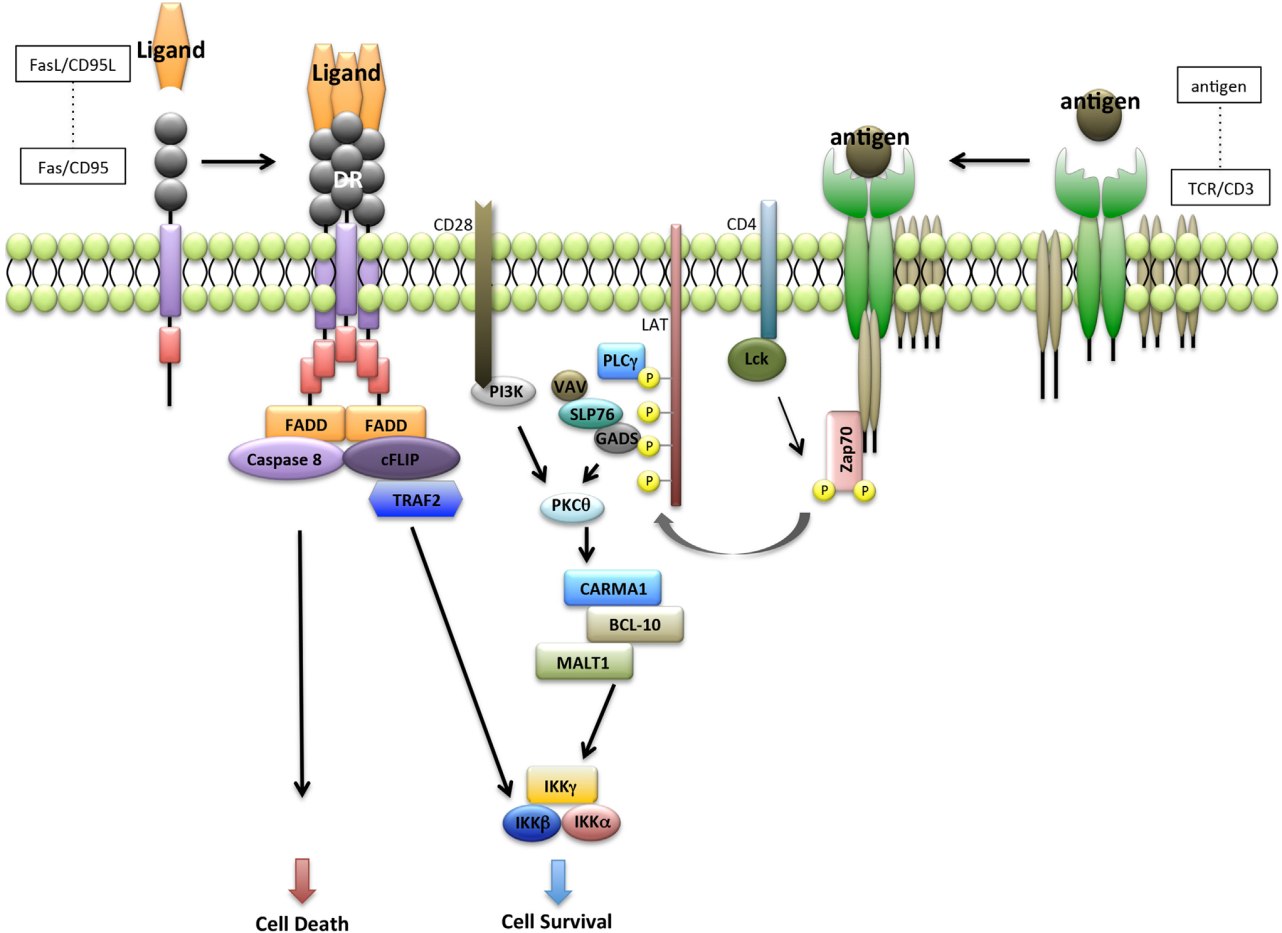
**Interaction between Fas receptor and T-cell receptor (TCR) pathway**. Fas-mediated apoptosis is initiated by the recruitment of Fas-associated protein with DD (FADD). FADD then recruits caspase-8 and forms a homodimer with caspase-8. Caspase-8 homodimers lead to the activation of caspase-3 and induce apoptosis. FADD also recruits caspase-8-like inhibitory protein (cFLIP) and form a heterodimer with caspase-8. cFLIP interacts with the IKK complex leading to nuclear factor-kappa β (NF-κB) activation. By contrast, ligation of TCR leads to recruitment of LCK and activation of ζ-chain-associated protein kinase 70 (ZAP70), which in turn induces tyrosine phosphorylation of linker for activation of T cells (LAT) and SH2-domain-containing leukocyte protein 76 (SLP76). SLP76 also induces the formation of complex that contains CARD-MAGUK protein-1 (CARMA1), BCL-10, and MALT1. BCL-10 and MALT1 are associated with active caspase-8 and cFLIP to promote the induction of antiapoptotic NF-κB target genes.

Following TCR ligation, signaling through CD3 complex leads to recruitment of signaling molecules such as lymphocyte-specific protein tyrosine kinase (LCK), phosphorylation of ζ-chain-associated protein kinase 70 (ZAP70), linker for activation of T cells (LAT), phospholipase Cγ1, VAV, SH2-domain-containing leukocyte protein 76, and protein kinase Cθ, in coordination with costimulatory molecules such as CD28 and CD4 (35, 36) (Figure 2).

Homeostasis of peripheral T cells is maintained by three mechanisms as follows: unresponsiveness (anergy) of T cells, suppression by regulatory T cells, and AICD (37). AICD is initiated by repeated stimulation of the TCR through Fas-mediated apoptosis to control the effector T-cell population (38). Further, AICD is induced through the interaction between Fas and FasL, and activated T cells expressing Fas and FasL are killed either though suicide or by mutual interaction (39, 40). During the immune responses, antigen-stimulated effector T cells are activated to produce various inflammatory cytokines and growth factors. Although the tuning of TCR signaling is controlled by costimulatory molecules such as CD28 and programmed cell death protein-1 (41), the system that declines the activated T cells maintains peripheral tolerance. Overactivated effector T cells are harmful to the immune system, and should be deleted from the periphery. Therefore, AICD induced by Fas-mediated apoptosis plays a potent role in the peripheral immune system (37).

Moreover, impairment of AICD contributes to the onset or development of autoimmunity. Numerous studies of *lpr/lpr* or *gld/gld* mice focused on the relationship between AICD of peripheral T cells and autoimmunity (10, 11, 19, 20). These studies found that impaired AICD in *lpr/lpr* or *gld/gld* mice leads to T cell dysfunctions and the onset of autoimmune lesions in multiple organs (10, 11, 19, 20). Evidence indicates that autoreactive T cells are abundant in the periphery of *lpr/lpr* or *gld/gld* mice as well as in patients with ALPS (10, 11, 19, 20). By contrast, Fas-independent T cell apoptosis is induced by the direct interaction between TRAIL-R2 on T cells and TRAIL on Fas-deficient dendritic cells in MRL-*lpr/lpr* mice (42). Thus, member of the TNFR family, including Fas and TRAIL-R2, likely play a key role in the maintenance of peripheral immune tolerance.

By contrast, Fas mediates apoptotic and non-apoptotic pathways (28). Acting downstream of cellular caspase-8-like inhibitory protein (cFLIP) and TRAF2 in the Fas signaling pathway, T-cell proliferation is induced through NF-κB activation (43) (Figure 2). The cFLIP N-terminal cleavage products p43-FLIP and p22-FLIP induce NF-κB activation by binding to the IKK complex (44, 45) (Figure 2). Further, overexpression of cFLIP inhibits Fas-induced apoptosis of activated T cells (46, 47). Moreover, Fas signaling regulates peripheral T cell homeostasis by modulating the equilibrium between proliferation and cell death, for example, in naive and memory T cell subsets (48). Therefore, homeostasis of peripheral T cells may be maintained by the dual outocomes of FasL/Fas signaling.

## Fas-Mediated B Cell Immune Regulation

The first report of the expression of FasL on the surface of B cells of wild-type mice but not from *gld/gld* mice demonstrated that activation of mouse B cells leads to the expression of FasL and killing of Fas-expressing target cells by B cells (49). Further, surface-localized FasL is expressed by human and mouse B cells, and the abnormal function of FasL^+^ killer B cells leads to a novel target for immune modulation in many disease settings (50–52). Moreover, reduction of the number of FasL^+^CD5^+^ B cells is associated with exacerbated severity of arthritis and inhibits T-cell death in a TCR transgenic mouse model with collagen-induced arthritis (53). Deficiency of the FasL/Fas signaling pathway in humans leads to ALPS, which is most often manifested as autoimmune hemolytic anemia, thrombocytopenia, or leukocytopenia, caused by cell-type specific autoantibody production (54, 55). Thus, FasL/Fas interactions play a critical role in regulating the production of pathogenic autoantibodies. The killer B cells that produce these autoantibodies may represent a novel modality for inducing T-cell death to treat autoimmunity.

Moreover, the expression of FasL on B cells of individuals infected by human immunodeficiency virus (HIV) (56) and the induction of Epstein–Barr virus of FasL expression by B cells markedly increases the sensitivity of Th cells to Fas-mediated T cell apoptosis (57). Although these data suggest that FasL expression by B cells might play a pathogenic role by inducing T cell death during viral infections, this hypothesis was not explored.

Findings that FasL^+^ B-chronic lymphocytic leukemia (B-CLL) cells kill a susceptible CD4^+^ T-cell leukemia cell line provided the first direct evidence implicating B cells in the induction of T cell apoptosis (52). FasL expression frequently occurs in patients with the aggressive form of B-CLL as well as in other human B-cell leukemias and lymphomas, most notably, multiple myeloma (58, 59). These results suggest that killer B cells induced by viral infections and tumor cells play a crucial role in the ability of virus-infected or cancer cells to evade the host’s immune system.

By contrast, Fas is highly expressed in activated and germinal center (GC) B cells. B cell-specific Fas deficiency is associated with the onset of autoimmunity (60), suggesting that Fas prevents the development of self-reactive GC B cells that escape normal regulatory controls and produce high amounts of immunoglobulin and autoantibodies (60, 61). Further, MRL-*lpr/lpr* mice produce circulating autoantibodies, such as rheumatoid factor as well as those against a single- and double-strand DNA and nuclear antigens (62, 63), indicating the contribution of FasL/Fas signaling in B cells to cellular processes such as differentiation, proliferation, death, and immunoglobulin production. Thus, Fas signal in B cell plays potent roles in the function and the pathogenesis of immune disorders.

## Unique Roles of FasL

FasL is a homotrimeric membrane protein that belongs to the TNF superfamily including CD178, CD95L, and apoptosis antigen-1 (Apo-1) ligand. Human FasL shares 81 and 78% amino acid sequence identity with its mouse and rat homologs, respectively. FasL comprises a single transmembrane domain, an intracellular domain harboring a proline-rich domain, and an extracellular domain. The latter contains an oligomerization domain required for oligomerization (64).

FasL expression on activated T cells is induced by stimulation *via* TCR, costimulatory molecules, and cytokine receptors (65). FasL expression is regulated by several transcription factors, including NF-κB, nuclear factor of activated T cells (NF-AT), early growth response gene family transcription factors, c-Myc, AP-1, secretory protein-1, and interferon regulatory factors (66–72).

Matrix metalloproteinase-7 (matrilysin) cleaves FasL to generate sFasL, which is released into the extracellular milieu (73). Abnormal levels of trimeric sFasL (73, 74) are detected in sera from patients with large granular lymphocytic leukemia and natural killer (NK) cell lymphoma (3), and other cancers as well as patients with SLE (75). The level of serum sFasL correlated with disease progression (3, 75) suggesting that sFasL induces the apoptosis of Fas^+^ T cells to evade immune surveillance. Thus, sFasL possesses proapoptotic and antiapoptotic properties depending of cell type and the microenvironment.

**Figure 3:**
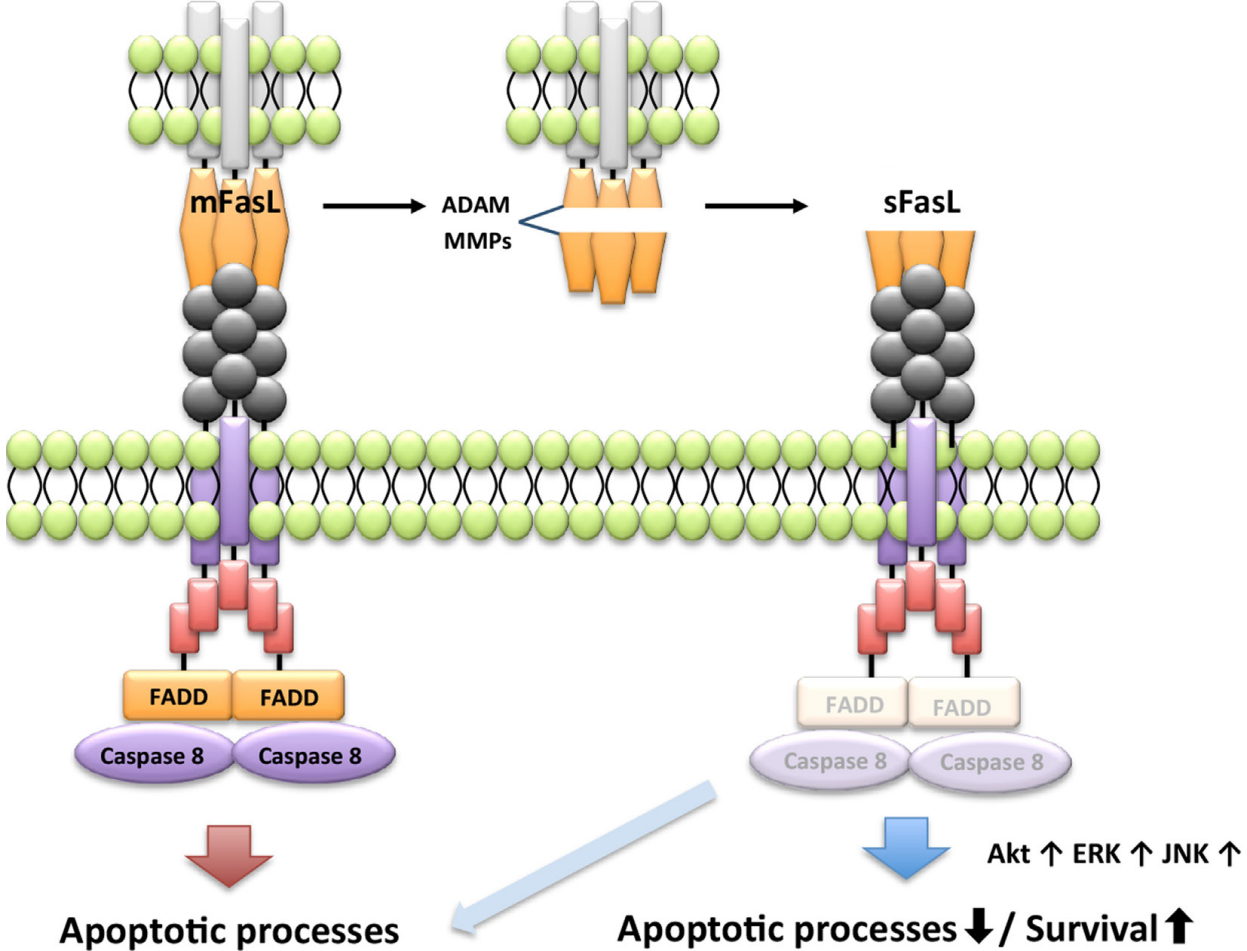
**Differential signaling pathway by membrane-bound FasL (mFasL) and soluble FasL (sFasL)**. mFasL leads to the recruitment of FADD and activates caspase pathways. mFas promotes apoptosis. mFasL is shed by a disintegrin and metalloproteinases (ADAM) or MMPs in the pathological or physiological condition. For instance, sFasL strongly activates extracellular signal-regulated kinase (ERK), PI_3_K/Akt, and c-jun N-terminal kinase (JNK) to induce proliferation and inhibit apoptotic activity of the fibroblast-like synoviocytes. Differential signal by the engagement of sFasL is determined by the cell type and the pathologic condition.

Apoptosis induced by FasL requires extensive oligomerization of the Fas receptor to activate the DISC (76), and membrane-bound FasL (mFasL) and sFasL can bind the Fas receptor. However, the naturally cleaved form of sFasL does not form oligomers with the Fas receptor, and therefore sFasL fails to induce apoptosis. Further, sFasL inhibits mFasL-mediated apoptosis through steric hindrance of its binding to the Fas receptor, indicating that mFas and sFasL have opposite functions that affect cell survival (77–79). For example, sFasL shed by a disintegrin and metalloproteinases or MMPs induces proliferation of fibroblast-like synoviocytes in patients with RA through activation of v-akt murine thymoma viral oncogene homolog (Akt1), extracellular signal-regulated kinase, and JNK (Figure 3) (80). Thus, sFasL has dual functions for cell death and survival.

Loss-of-function mutations in the genes encoding murine and human FasL causes a phenotype similar to that of patients with lymphadenopathy and autoimmune disease because of decreased apoptosis in Fas^+^CD4^−^CD8^−^ T lymphocytes and the production of autoantibodies (81, 82). In patients with ALPS, germline mutations of FasL gene are associated with defective apoptosis (83). Patients with rare heterozygous FasL gene mutations are classified as type Ib-ALPS (84).

The tissue distribution of FasL, which is predominantly expressed by activated T cells and NK cells, is limited (3). FasL is constitutively expressed by stromal cells of the retina and Sertoli cells of the testis, respectively, which are immunoprivileged tissues. In such tissues, FasL expression leads to the death of invading Fas^+^ cells. The susceptibility of the eyes of *gld/gld* mice to inflammation (85) and their rejection of corneal allografts prove that FasL plays an important role in sites of immune privilege (24). The ocular immune privilege is believed to be one mechanism by which the visual axis is protected from dangerous immune reactions. Therefore, immunoprivileged site is maintained by the sequestration of any antigens, the lack of lymphatic damage, and the blood-tissue barrier (5). In addition, both Fas and FasL are present on thyroid cells in patients with autoimmune thyroiditis (Hashimoto’s thyroiditis) (22). This suggests that Fas/FasL interactions among thyroid cells contribute to the pathogenesis of autoimmune thyroiditis with tissue destruction (22).

FasL is expressed in tumors such as colorectal carcinomas, melanomas, head and neck carcinomas, and myelomas (86). The level of FasL produced by non-immune cells can induce apoptosis in Fas^+^ T cells that recognize tissue-specific antigens to evade immune surveillance (86). Moreover, a variety of cell types can express FasL in response to different stimulatory conditions, including macrophages infected with HIV, hepatocytes treated with ethanol, leukemia cells exposed to chemotherapy drugs, as well as cancer cells (87, 88). Thus, the unique functions of FasL contribute to various physiological or pathological processes that are not associated with the immune system.

## The Fas/FasL System in Autoimmunity

The contribution of Fas-mediated apoptosis to the onset and development of autoimmunity was established by studies of patients with autoimmune diseases and animal models (14, 15, 89, 90). ALPS is an inherited disorder of the systemic immune system that involves a spontaneous mutation in the Fas or FasL gene (14, 15, 21). Approximately two-thirds of patients with ALPS bear mutations in the gene encoding Fas (type Ia) (91, 92). By contrast, there are few reports of mutations in the genes encoding FasL gene (type Ib) and caspase-10 gene (ALPS type IIa) (92–95), and these mutations are not detected in patients with ALPS type III (96).

The clinical features of patients with ALPS are splenomegaly, lymphadenopathy, and hepatomegaly, which are caused by the accumulation of polyclonal lymphocytes as well as autoimmune lesions in multiple organs. The risk of malignant lymphoma is increased in patients with ALPS (97). Peripheral T cells expressing TCRα/β, but not CD4 and CD8 [CD4^−^CD8^−^ double negative (DN)], proliferate, and their population expands in the patients with ALPS due to an impaired AICD caused by a defect in the Fas/FasL system (98). Further, the abnormal programming of Fas-deficient T cells before the DN T-stage is caused by impaired signaling through the mTOR pathway (99). Moreover, a defect in B-cell selection occurs in patients with ALPS, which is caused by impaired class-switch recombination and somatic hypermutation of the genes encoding immunoglobulins (100). Polymorphisms in the genes encoding Fas and FasL are associated with the susceptibility and severity of autoimmune lesions in patients with RA (101, 102) as well as in patients with primary SS (103).

To understand the mechanism of Fas/FasL-mediated apoptosis *in vivo*, MRL-*lpr/lpr* mice were used as a model of susceptibility to autoimmune disease before spontaneous mutation of the gene encoding Fas was discovered to affect the onset of autoimmunity in the mice (10). Further, *gld/gld* mice bearing a spontaneous mutation of the gene encoding FasL are employed as a model for autoimmune disease as well (19, 20). Lymphoproliferative lesions in *lpr/lpr* and *gld/gld* mice demonstrate that Fas/FasL-mediated apoptosis plays a critical role in controlling the maintenance of peripheral lymphocytes. In particular, Fas/FasL-mediated apoptosis contributes to the AICD of T cells in the periphery (4). Moreover, peripheral T cell apoptosis is induced by FasL expression by macrophages, and apoptotic T cells are promptly phagocytosed by these macrophages, depending on their level of Fas expression (104). In these experiments, normal T cells of C57BL/6 (B6) mice were engulfed by macrophages of B6-*lpr/lpr* mice, which lead to enhanced Fas expression by donor T cells through IFN-γ/IFN-γ-receptor signaling (104). These findings revealed that the control of Fas expression by macrophages plays an essential role in maintaining T cell homeostasis in the peripheral immune system.

By contrast, the expression of Fas or FasL occurs in target organs of patients with autoimmune diseases and in animal models (105, 106). High levels of sFasL are present in the synovial fluid of patients with RA (107), which may be associated with Fas-mediated apoptosis of synovial cells, but not the AICD of T cells. Further, sFasL present in the synovial fluid of patients with RA inhibits angiogenesis in RA lesions (108). These observations are consistent with decreased levels of FasL mRNA expression in lacrimal gland tissues and peripheral blood lymphocytes as well as increased levels of Fas and FasL in salivary gland tissues of patients with SS (109, 110). These findings implicate Fas-mediated apoptosis in the destruction of target salivary gland tissue.

When we treated SS model mice with an anti-murine FasL-specific monoclonal antibody to protect against Fas-mediated apoptosis of the target salivary gland cells (111), we unexpectedly observed exacerbation of the autoimmune lesions in the salivary and lacrimal glands (111). We found that sFasL is processed by autoantigen-specific CD4^+^ T cells concomitant with metalloproteinase-9 expression (65, 111), indicating that increased sFasL expression inhibits the normal AICD of T cells and leads to the proliferation of autoreactive T cells in this SS model. Moreover, although mFasL, but not sFasL, is essential for cytotoxic activity that guards against lymphadenopathy and autoimmunity (29), it remains unclear whether the relationship between mFasL and sFasL contributes to the molecular mechanisms of AICD. Thus, the impairment of Fas/FasL system in the peripheral immune tolerance considerably contributes to the onset or development of a lot of autoimmune diseases.

## Concluding Remarks

Some examples regarding the relationship between various cells and Fas/FasL expression are listed in Table 1. Numerous molecules precisely regulate Fas-mediated apoptosis through complicated signaling cascades. Apoptotic and antiapoptotic signaling pathways in T cells are controlled by the interaction between TCR and Fas signaling pathways and the expression of FasL. Further, the Fas/FasL system plays potent roles in B cell biology. The unique functions of FasL contribute to tumorgenesis, infection, immune disorders as well as to the outcomes of tissue transplantation. Finally, the multiple functions of Fas/FasL-mediated regulation maintain immune tolerance.

**Table 1:** **Relationship between cell type and Fas/FasL expression**.

Cell type	Fas/FasL expression	Remarks
T cell	Fas^+^, FasL^+^	Activation-induced cell death, autoimmune lymphoproliferation syndrome (ALPS), autoimmunity
B cell	Fas^+^, FasL^+^	ALPS, collagen-induced arthritis, virus infection, germinal center, B-chronic lymphocytic leukemia
Natural killer cell	FasL^+^	Tumor immunity
Macrophage	Fas^+^, FasL^+^	Peripheral immune tolerance
Thyrocyte	Fas^+^, FasL^+^	Hashimoto’s thyroiditis, immune privilege
Sertoli cell	FasL^+^	Immune privilege
Salivary gland cell	Fas^+^	Sjögren’s syndrome
Corneal cell	FasL^+^	Immune privilege
Synovial cell, fluid	Fas^+^, soluble FasL	Rheumatoid arthritis

## Author Contributions

All authors contributed to the writing of the manuscript. NI conceived and edited the manuscript. YK and MS prepared the figures.

## Conflict of Interest Statement

The authors declare that the research was conducted in the absence of any commercial or financial relationships that could be construed as a potential conflict of interest.

## Abbreviations

AICD, activation-induced cell death; ALPS, autoimmune lymphoproliferation syndrome; TNF-α, tumor necrosis factor-alpha; TRAIL, TNF-related apoptosis-inducing ligand; TRAF2, TNF receptor-associating factor 2; TCR, T-cell receptor; cFLIP, caspase-8-like inhibitory protein, NF-κB, nuclear factor-kappa β.
